# Application of vagus nerve stimulation on the rehabilitation of upper limb dysfunction after stroke: a systematic review and meta-analysis

**DOI:** 10.3389/fneur.2023.1189034

**Published:** 2023-06-21

**Authors:** Xu Wang, Qixin Ding, Tianshu Li, Wanyue Li, Jialin Yin, Yakun Li, Yuefang Li, Weisheng Zhuang

**Affiliations:** ^1^School of Rehabilitation Medicine, Henan University of Chinese Medicine, Zhengzhou, China; ^2^School of Clinical Medicine, Henan University, Zhengzhou, China; ^3^Department of Rehabilitation Medicine, The First Affiliated Hospital of Jinan University, Guangzhou, China; ^4^Department of Rehabilitation, Henan Provincial People's Hospital, School of Rehabilitation Medicine, Henan University of Chinese Medicine, Zhengzhou, China

**Keywords:** vagus nerve stimulation (vns), stroke, rehabilitation, meta-analysis, upper limb dysfunction

## Abstract

**Objective:**

This study aimed to elucidate the efficacy, safety, and long-term implications of vagus nerve stimulation (VNS) as a viable therapeutic option for patients with upper limb dysfunction following a stroke.

**Methods:**

Data from the following libraries were searched from inception to December 2022: PubMed, Wanfang, Scopus, China Science and Technology Journal Database, Embase, Web of Science, China Biology Medicine Disc, Cochrane Library, and China National Knowledge Infrastructure. Outcomes included indicators of upper limb motor function, indicators of prognosis, and indicators of safety (incidence of adverse events [AEs] and serious AEs [SAEs]). Two of the authors extracted the data independently. A third researcher arbitrated when disputes occurred. The quality of each eligible study was evaluated using the Cochrane Risk of Bias tool. Meta-analysis and bias analysis were performed using Stata (version 16.0) and RevMan (version 5.3).

**Results:**

Ten trials (VNS combined with rehabilitation group vs. no or sham VNS combined with rehabilitation group) with 335 patients were included in the meta-analysis. Regarding upper extremity motor function, based on Fugl–Meyer assessment scores, VNS combined with other treatment options had immediate (mean difference [MD] = 2.82, 95% confidence interval [CI] = 1.78–3.91, *I*^2^ = 62%, *p* < 0.00001) and long-term (day-30 MD = 4.20, 95% CI = 2.90–5.50, *p* < 0.00001; day-90 MD = 3.27, 95% CI = 1.67–4.87, *p* < 0.00001) beneficial effects compared with that of the control treatment. Subgroup analyses showed that transcutaneous VNS (MD = 2.87, 95% CI = 1.78–3.91, *I*^2^ = 62%, *p* < 0.00001) may be superior to invasive VNS (MD = 3.56, 95% CI = 1.99–5.13, *I*^2^ = 77%, *p* < 0.0001) and that VNS combined with integrated treatment (MD = 2.87, 95% CI = 1.78–3.91, *I*^2^ = 62%, *p* < 0.00001) is superior to VNS combined with upper extremity training alone (MD = 2.24, 95% CI = 0.55–3.93, *I*^2^ = 48%, *p* = 0.009). Moreover, lower frequency VNS (20 Hz) (MD = 3.39, 95% CI = 2.06–4.73, *I*^2^ = 65%, *p* < 0.00001) may be superior to higher frequency VNS (25 Hz or 30 Hz) (MD = 2.29, 95% CI = 0.27–4.32, *I*^2^ = 58%, *p* = 0,03). Regarding prognosis, the VNS group outperformed the control group in the activities of daily living (standardized MD = 1.50, 95% CI = 1.10–1.90, *I*^2^ = 0%, *p* < 0.00001) and depression reduction. In contrast, quality of life did not improve (*p* = 0.51). Safety was not significantly different between the experimental and control groups (AE *p* = 0.25; SAE *p* = 0.26).

**Conclusion:**

VNS is an effective and safe treatment for upper extremity motor dysfunction after a stroke. For the functional restoration of the upper extremities, noninvasive integrated therapy and lower-frequency VNS may be more effective. In the future, further high-quality studies with larger study populations, more comprehensive indicators, and thorough data are required to advance the clinical application of VNS.

**Systematic review registration:**

https://www.crd.york.ac.uk/prospero/, identifier: CRD42023399820.

## 1. Introduction

Stroke is a severe health risk that represents a great burden to society and healthcare systems. Approximately 60% of the individuals who experience a stroke have long-lasting upper extremity dysfunction that hinders their activities of daily living and compromises their mental wellbeing (1–4). It has been predicted that, by 2050, there will be ~200 million stroke victims worldwide. Hence, it is paramount that more effective treatment strategies are developed (5). Alternative approaches are required because standard rehabilitation therapy may not successfully restore function after a stroke (6). Ideally, future therapies for stroke should combine thrombolysis with antithrombotic, neuroprotective, and neuroplasticity-enhancing interventions (7). One possible treatment for enhancing neuroplasticity of the upper limb following a stroke is vagus nerve stimulation (VNS). VNS is an adjunctive therapy approved by the Food and Drug Administration for the treatment of partial epilepsy, depression, and primary headache disorders (8). VNS refers to any method that stimulates the vagus nerve. The methods are divided into invasive VNS (iVNS) and transcutaneous VNS (tVNS). Furthermore, tVNS can be further divided into transcutaneous cervical VNS (tcVNS) and transcutaneous auricular VNS (taVNS) (9, 10). The number of publications related to VNS has tripled in the last 10 years. In particular, the number of published studies has exponentially increased over the last few years (11). Numerous preclinical studies have documented positive poststroke recovery following a combination of VNS and physical therapy. Numerous clinical studies have also produced encouraging findings (12).

Animal studies involving rats with cerebral ischemia have suggested that VNS combined with rehabilitation can significantly alleviate neurological impairment, reduce cerebral infarction volume, and improve forelimb function, as well as memory and cognition (13–17). The mechanism of action may include enhancing angiogenesis, controlling blood-brain barrier permeability, minimizing the spread of depolarization, preventing neuroinflammation, and facilitating poststroke axonal plasticity (18–22). An increasing number of clinical trials have also demonstrated the beneficial effects of VNS combined with rehabilitation for patients with stroke. However, most clinical trials have been limited by small sample sizes (23, 24). Several meta-analyses have concluded based on the available clinical trials that VNS may improve the recovery of upper limb function following a stroke (25–32). Additionally, some researchers have reported that tVNS may be more effective than iVNS (26, 28, 29, 31, 32). These published meta-analyses had a risk of publication bias due to the absence of funnel plots. Many of these meta-analyses have also highlighted the need for more welldesigned studies to verify the long-term efficacy of VNS, including the stimulation settings, prognostic scores, integrated rehabilitation training methods, adverse events (AEs), and other factors. The analyses conducted by Liu et al. (28) and Zhao et al. (32) also restricted the studies to specific languages. In light of the potential clinical significance of VNS and the currently weak evidence from quantitative analyses, this study aimed to conduct a comprehensive and up-to-date meta-analysis on VNS for upper limb dysfunction after a stroke, including the efficacy, safety, and long-term implications.

## 2. Data and methods

This study was a systematic review of previously published studies. Therefore, both patient consent and ethical approval were not required (33). The meta-analysis was conducted according to the Preferred Reporting Items for Systematic Reviews and Meta-Analyses guidelines and previously published protocols (34). The detailed protocol used to perform this systematic evaluation has been registered in PROSPERO (reference number: CRD42023399820).

### 2.1. Search strategy

The following databases were searched from the time of inception to December 2022: PubMed, Scopus, Embase, Web of Science, Cochrane Library, China National Knowledge Infrastructure, Wanfang, China Science and Technology Journal Database, and China Biology Medicine. The following search terms were used: (Stroke OR Cerebrovascular Accident OR CVA OR Cerebrovascular Apoplexy OR Vascular Accident OR Cerebral Stroke) AND (VNS OR Vagal Nerve Stimulation OR Vagal Nerve Stimulation). To identify further relevant articles, we traced the references included in the identified articles and conducted manual searches.

### 2.2. Inclusion and exclusion criteria

We searched for studies without language restrictions. The inclusion criteria were as follows: (1) Studies with patients with stroke and upper limb disorders; (2) the experimental group received VNS combined with other treatment approaches, and the control group received no VNS or sham stimulation combined with other treatment approaches. The other treatment approaches were the same in the experimental and control groups; (3) studies that were randomized controlled trials (RCT); and (4) the study included at least one of the following pretraining or follow-up outcome indicators: motor function, quality of life, activities of daily living (ADL), and/or AEs. The exclusion criteria were as follows: (1) Patients who experienced a non-primary stroke; (2) relevant data required for meta-analysis were not available; (3) the full text of the paper could not be obtained even after contacting the corresponding author.

### 2.3. Data extraction

The following data were gathered: author, location, publication year, disease course, disease type, the number of samples, intervention modes, the type of combined therapy, stimulus parameters, stimulus time, evaluation time, and outcomes. Two researchers independently screened the papers and extracted and crosschecked the data. Any dispute was resolved through discussion or negotiation with an independent researcher. We used the Java program GetData Graph Digitizer (http://www.getdata-graph-digitizer.com) to determine the numerical values from the plotted data if no values were originally provided. If there were no pre- and post-treatment differences in the included randomized controlled trials or post-treatment data, the corresponding author was contacted to obtain the missing details. Where necessary, we manually calculated the mean and standard deviation (SD) using the Cochrane Handbook formulas based on the available baseline and outcome data.

### 2.4. Outcome measures

The outcome measures in this study were the efficacy and safety of VNS for the treatment of upper limb dysfunction after a stroke. Efficacy referred to the improvement of upper limb motor function and its impact on patient prognosis, while safety included the number of AEs and serious AEs (SAEs). The main indicator of upper limb motor function was the Fugl–Meyer Assessment for Upper Extremity (FMA-UE) score after VNS treatment at different frequencies combined with different treatment methods. The secondary indicators were the Wolf Motor Function Test (WMFT) score and FMA-UE effective rate. The prognosis was defined as improvement in ADL, quality of life, and mental wellbeing (e.g., mood).

### 2.5. Quality assessment

The quality of all the articles was assessed independently by the two researchers who reviewed the findings. When a disagreement occurred, a third researcher was consulted for arbitration. The quality of the included RCTs was assessed using the Cochrane risk-of-bias tool (35). This involved evaluating seven different types of biases: attrition bias (incomplete outcome data), selection bias (unbiased sequence generation and allocation concealment), reporting bias (selective result reporting), blinding bias (unbiased performance and detection), and other bias. The risk of bias for each item was rated as low, unclear, or high.

### 2.6. Statistical analysis

The evaluation index data of the included studies were processed using RevMan software (version 5.3; Cochrane Collaboration, Software Update, Oxford, UK). The mean difference (MD) and 95% confidence interval (CI) were used to express continuous variables. For continuous variables with different units, the standardized MD (SMD) and 95% CI were applied to exclude the influence of units (36). Dichotomous variables were expressed as risk ratios using the Mantel–Haenszel method. The degree of study heterogeneity was represented using *I*^2^. A random-effects model was applied if *I*^2^ exceeded 50%. Otherwise, a fixed-effects model was used. Values >75% indicated high heterogeneity. Sensitivity and subgroup analyses were utilized to pinpoint the source of heterogeneity and also to examine the stability of the results, as well as compare the effects of different clinical factors. Descriptive analysis was performed if the cause of the heterogeneity was not identified. Stata software (version 16.0, http://www.stata.com) was used to construct a funnel plot to determine publication bias. Finally, we used GRADE profiler software (https://gradeprofifiler.software.informer.com/) to evaluate the quality of the evidence based on the analyzed outcome indicators of the present study.

## 3. Results

### 3.1. Search results, study characteristics, and quality assessment

**Figure 1:**
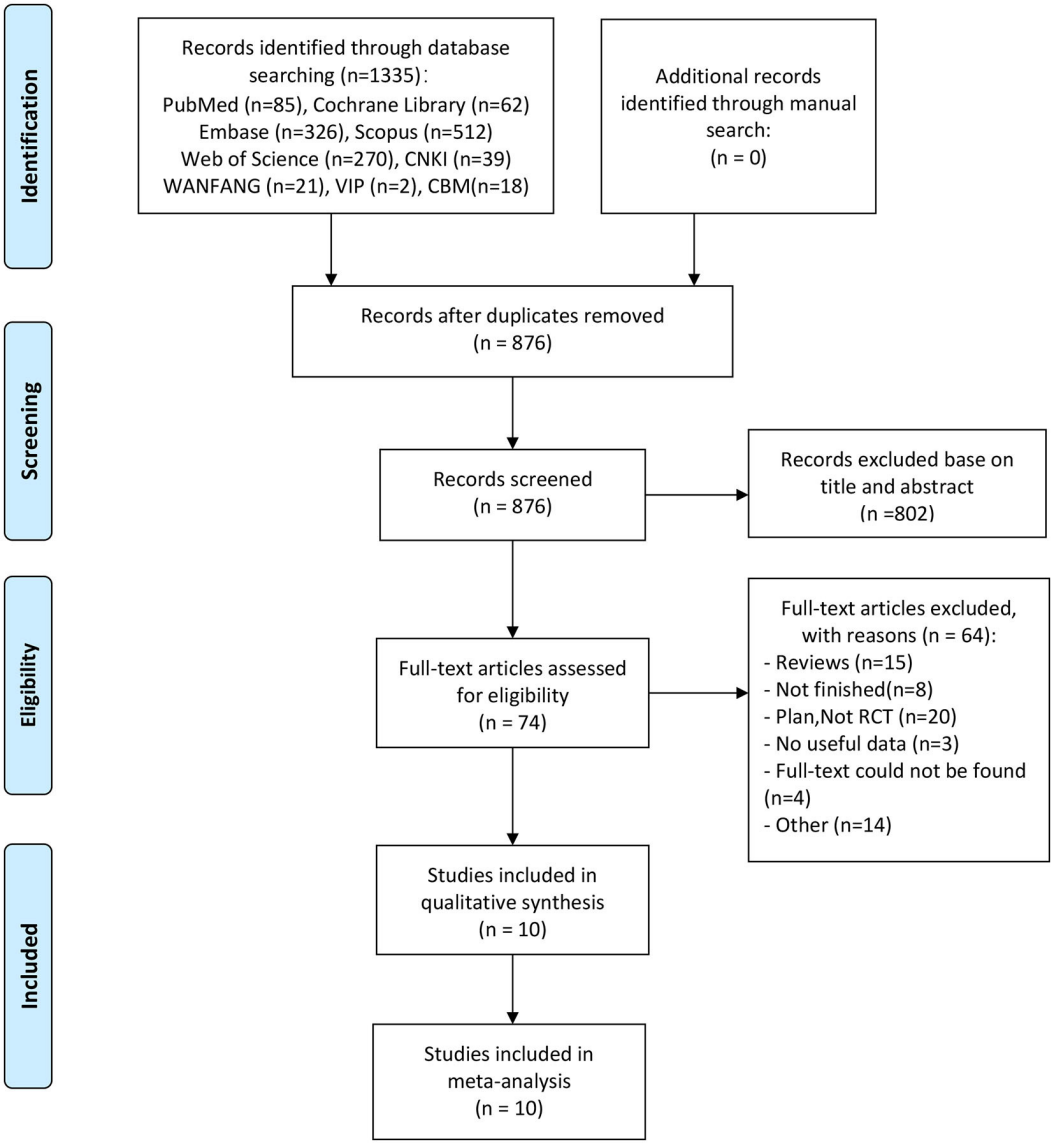
The flowchart of the literature search and screening process.

**Figure 2:**
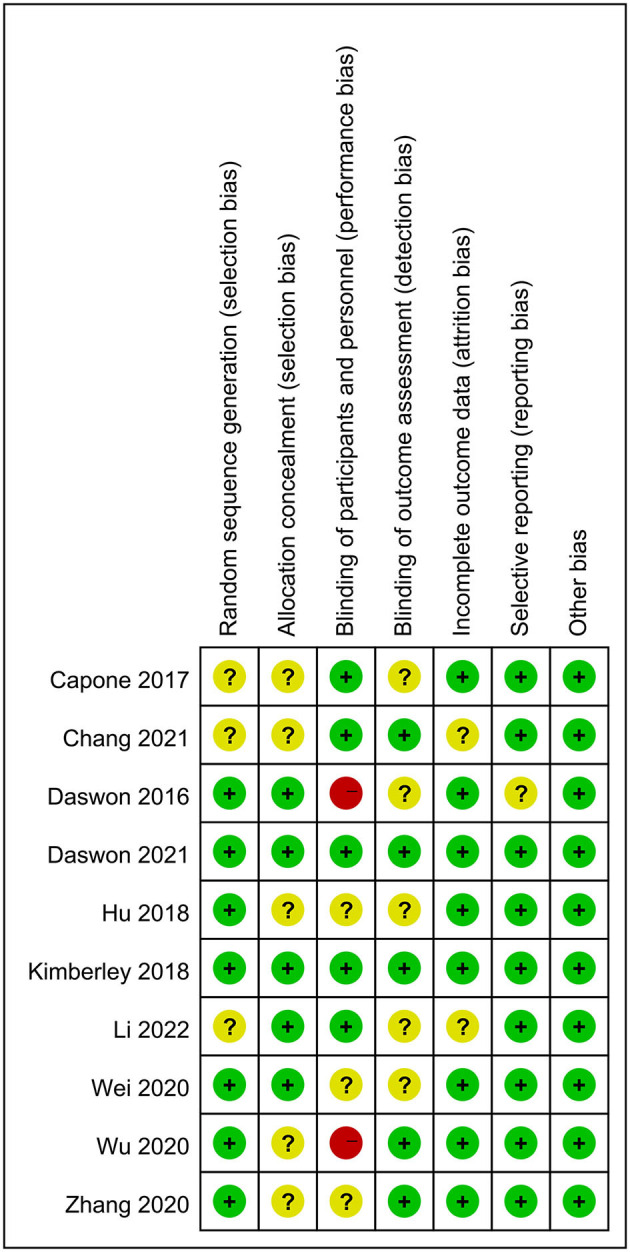
Risk of bias summary of RCTs. RCT, Randomized controlled experiment.

The flowchart of the search and article selection process is shown in Figure 1. Initially, 1,335 articles were identified as potentially relevant. Ten articles (three written in Chinese and seven in English) (23, 24, 37–44), involving 335 participants, were finally included in this study. The basic details of the 10 included articles are shown in Table 1. All 10 articles were RCTs with an experimental group and a control group. The experimental groups underwent VNS with different stimulus parameters combined with other treatment approaches. Eight of the 10 studies utilized a placebo in the control group, while the other two were blank controls (no VNS) (Table 1). The combined treatment methods employed with VNS included upper limb therapy alone in six studies and comprehensive therapy in three studies. The interventions lasted for 2–4 weeks, and evaluations were performed 1–90 days after the treatment. Wu et al. (41) and Dawson et al. (23) did not blind the outcome assessments (detection bias), but the remaining eight studies were blinded (Figure 2).

**Table 1:** Basic information of the included studies.

**Author year**	**Location**	**Disease**	**Type**	**N (E/C)**	**Mode (E/C)**	**Combined therapy**	**Stimulus parameters and time**	**Evaluation time (day)**	**Effective index**	**Safe index**
Dawson et al. (26)	England 2 centers	IC	>6 months	9/11	iVNS	Upper limb training	30 Hz, 0.1 ms, 0.8 mA, 0.5 s/10 s 120 min per day, 3 times per week, 6 w	1, 30, 90	FMA-UE, ARAT, grip strength, NHPT, Box and Block test	AE, SAE
Capone et al. (24)	Italy	IC/ICH	>1 year	7/5	taVNS/ sham	Upper limb robot training	20 Hz, 0.3 ms, patients' tolerance (1.1 ± 9.0 mA), 30 s/5 min, 60 min per day, 10 days	1	FMA-UE	sBP, dBP, HR
Kimberley et al. (39)	America	IC	4 months to 3 years	8/9	iVNS/ sham	Upper limb training	30 Hz, 0.1 ms, 0.8 mA, 0.5 s/10 s, 120 min per day, 3 times per week, 6 w	1, 30, 90	FMA-UE, WMFT, Nine-hole test, MAL, SIS	AE, SAE
Zhenguo (38)	China	NA	>2 months	40/40	VNS	Comprehensive therapy	3 months, NA	1	FMA, MBI, NIHSS	
Wu et al. (40)	China	IC	subacute	10/11	taVNS/ sham	Comprehensive rehabilitation training	20 Hz, 0.3 ms, patients' tolerance (1.66 ± 0.40 mA), 30 s/2 min 30 min per day, 10 consecutive days	1, 30, 90	FMA-UE, WMFT, FIM, Brunstrom	HR, sBP, dBP, AE
Wei et al. (30)	China	IC	2 weeks to 3 months	13/13	taVNS/sham	Upper limb training	25 Hz, 0.1 ms, patients' tolerance, 60 min per day, 5 times per week, 4 w	1, 30	FMA-UE, Brunstrom, MFAS, MAS, BI	HR, AE
Liping (37)	China	IC	>24 hours < 3 months	21/21	taVNS/ sham	Medical treatment and comprehensive rehabilitation training	20 Hz, 0.5 mA, 30 s/2 min 30 min per time, 5 times per week, 3 w	1	FMA-UE, WMFT, FIM	AE
Chang et al. (44)	America	NA	>6 months	14/15	taVNS/ sham	Upper limb robot training	30 Hz, 0.3 ms, patients' tolerance (0.1–0.5 mA), 0.5 s/10 s, 60 min per time, 3 times per week, 3 w	1	FMA-UE, MRC, WMFT	AE
Dawson et al. (43)	America, 19 centers	IC	>9 months	53/55	iVNS/ sham	Upper limb training	30 Hz, 0.1 ms, 0.8 mA, 0.5 s/10 s, 120 min per day, 3 times per week, 6 w	1, 90	FMA-UE, WMFT, MAL, SIS, SS-QOL, EQ-D, BDI	AE, SAE
Li et al. (6)	China	IC/ICH	< 1 month	28/28	taVNS/ sham	Comprehensive rehabilitation	20 hz, 0.3 ms, patients' tolerance (1.71 ± 0.5 mA), 30 s/5 min, 20 min per time, 5 times per week, 4 w	1	WMFT, FMA-UE, FMA-L, FMA-S, HADS, SIS	HR, sBP, dBP, AE

E, experiment group; C, control group; NA, no answer; IC, ischemic cerebral infraction; ICH, hemorrhage cerebral infraction; tVNS, transcutaneous vagus nerve stimulation; taVNS, transcutaneous auricular vagus nerve stimulation; tcVNS, transcutaneous cervical VNS; iVNS, invasive vagus nerve stimulation; FMA-UE, Fugl-Meyer Assessment for Upper Extremity; FMA-L, Fugl-Meyer Assessment for lower limb function; FMA-S, Fugl-Meyer Assessment for sensory; SIS, Stroke Impact Scale; ARAT, Action Research Arm Test; WMFT, Wolf Motor Function Test; MAL, Motor Activity Log; FIM, Functional Independence Measurement; EQ-D, EuroQol Five Dimensions Questionnaire; BDI, Beck Depression Inventory; HADS, Hospital Anxiety, and Depression Scale; SS-QOL, Stroke-Specific Quality of Life; BDI, Beck Depression Inventory; AE, adverse events; SAE, serious AEs; HR, heart rate; sBP, systolic blood pressure; dBP, diastolic blood pressure.

### 3.2. The efficacy of VNS used in stroke treatment

#### 3.2.1. The primary indicator of upper limb motor function measured by the FMA-UE score

**Figure 3:**
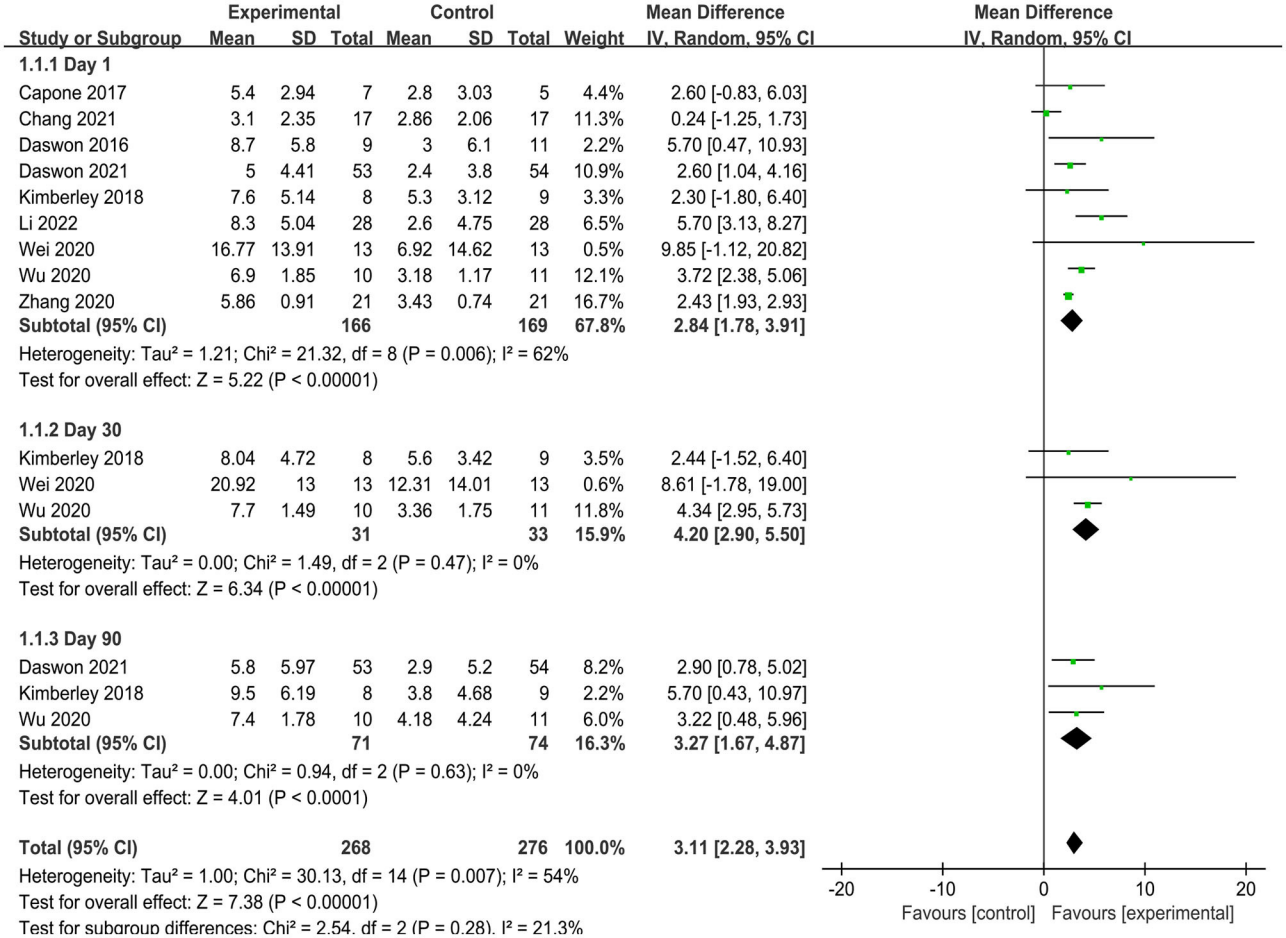
Frost plot of the immediate and long-term effects of FMA-UE.

Nine articles reported the FMA-UE scores a day after treatment (immediate effect). The available results indicated that the VNS group significantly improved upper limb motor function compared with the control group (MD = 2.84, 95% CI = 1.78–3.91, *I*^2^ = 62%, *p* < 0.00001; Figure 3) (23, 24, 37, 39–44). Three articles reported FMA-UE scores at 30- and 90-day posttreatment (long-term effects) (24, 41, 43). The pooled findings indicated that the scores in the VNS group were significantly higher than those in the control group at 30- (MD = 4.20, 95% CI = 2.90–5.50) and 90-day posttreatment (MD = 3.27, 95% CI = 1.67–4.87) (Figure 3). Based on these results, VNS demonstrated immediate and long-term effects on upper limb motor function.

**Figure 4:**
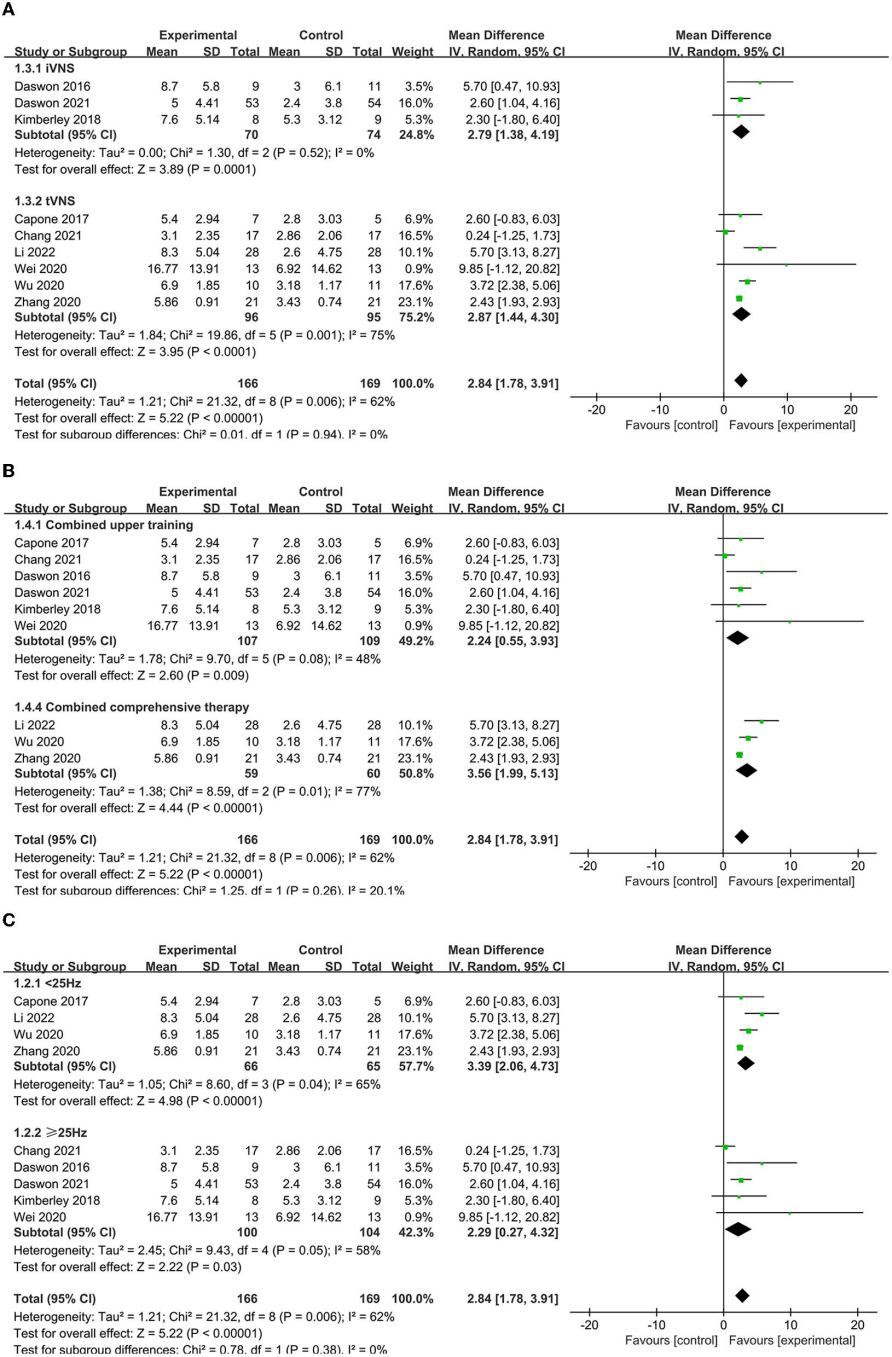
**(A)** Subgroup analysis of different modes of VNS in FMA-UE scores. **(B)** Subgroup analysis of VNS combined with different treatments. tVNS, transcutaneous vagus nerve stimulation; iVNS, invasive vagus nerve stimulation. **(C)** Subgroup analysis of VNS with different frequencies in FMA-UE scores.

A subgroup analysis was conducted to compare various aspects that may influence efficacy, such as disease stage, combination protocol, stimulation modality, and other stimulation parameters. The results revealed that tVNS (MD = 2.87, 95% CI = 1.78–3.91, *I*^2^ = 62%, *p* < 0.00001) may be superior to iVNS (MD = 3.56, 95% CI = 1.99–5.13, *I*^2^ = 77%, *p* < 0.0001, Figure 4A). Moreover, VNS in conjunction with combination therapy (MD = 2.87, 95% CI = 1.78–3.91, *I*^2^ = 62%, *p* < 0.00001) outperforms VNS in conjunction with upper extremity training alone (MD = 2.24, 95% CI = 0.55–3.93, *I*^2^ = 48%, *p* = 0.009, Figure 4B). Furthermore, lower frequency VNS (< 25 Hz) (MD = 3.39, 95% CI = 2.06–4.73, *I*^2^ = 65%, *p* < 0.00001) may be superior to higher frequency VNS (≥25 Hz) (MD = 2.29, 95% CI = 0.27–4.32, *I*^2^ = 58%, *p* = 0.03) (Figure 4C).

#### 3.2.2. Secondary indicators of upper extremity motor function measured by the FMA-UE efficiency and WMFT score

**Figure 5:**
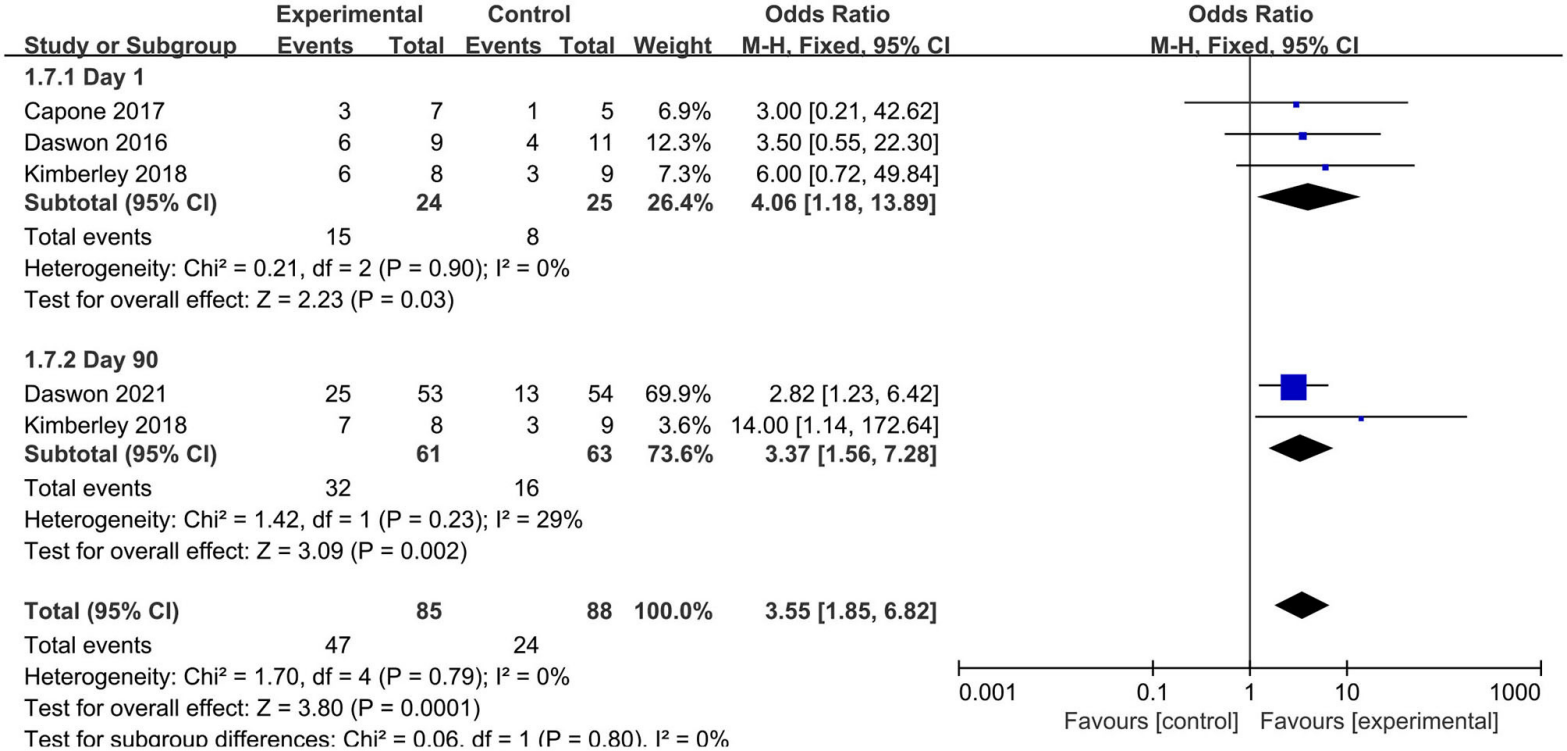
Forest plot of FMA-UE efficiency.

FMA-UE efficiency was defined as an increase in the FMA-UE score by >6. Three articles (23, 24, 39) reported the FMA-UE scores at 1 day posttreatment (immediate effect), and two articles (39, 43) reported the FMA-UE scores at 90 days posttreatment (long-term effect) (Figure 5). Pooled analyses indicated that both the immediate (MD = 4.06, 95% CI = 1.18–13.89) and long-term (MD = 3.37, 95% CI = 1.56–7.28) effects had little heterogeneity. The fixed-effects model was employed, and the results indicated that FMA-UE efficiency was higher in the experimental group than that in the control group.

**Figure 6:**
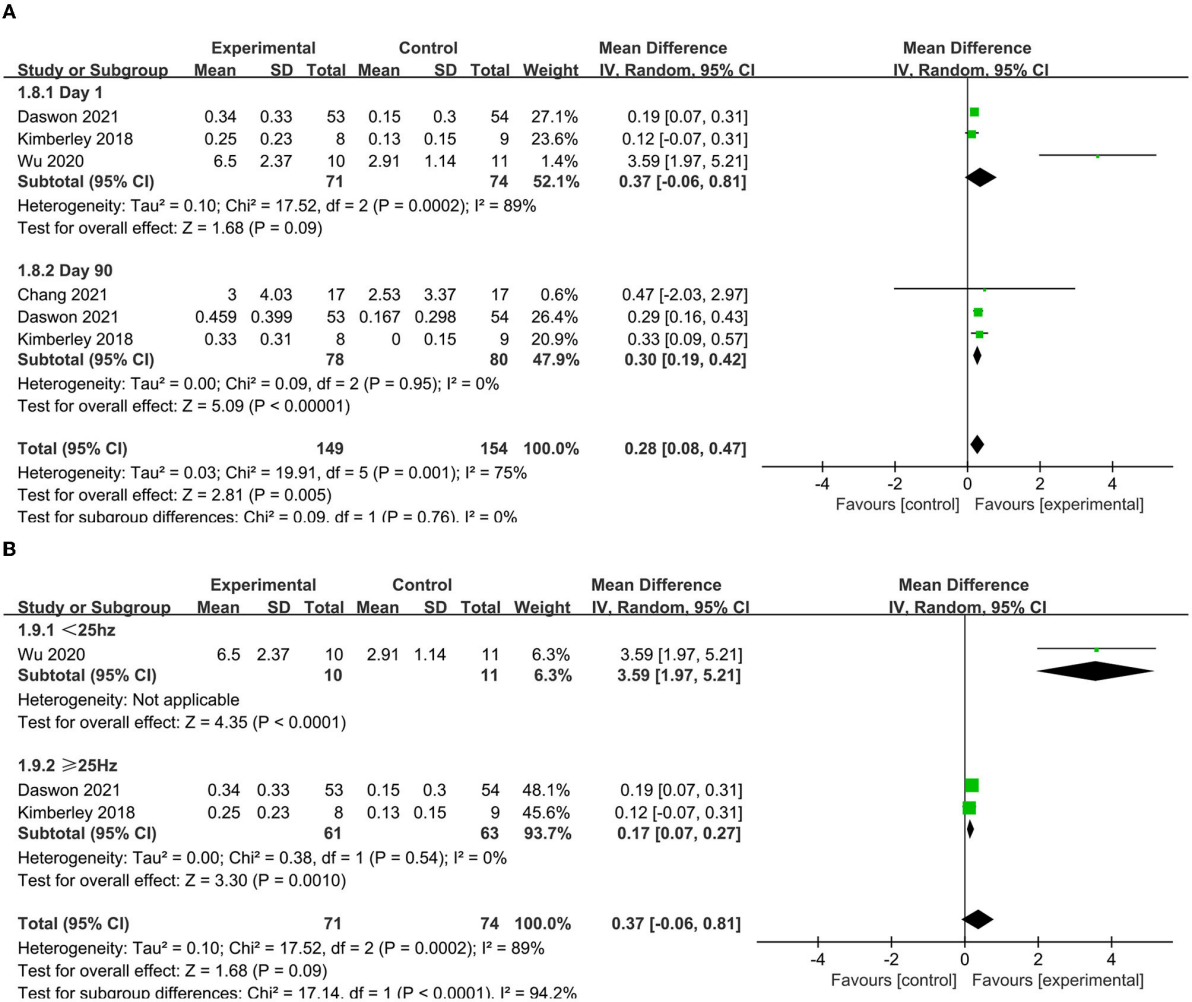
**(A)** Forest plot of WMFT. **(B)** Subgroup analysis of VNS with different frequencies in WMFT scores.

Three articles (39, 41, 43) reported the immediate effect as indicated by the WMFT score. The pooled analysis indicated that the WMFT score was higher in the experimental group than that in the control group (MD = 0.37, 95% CI = 0.06–0.81, *I*^2^ = 89%) (Figure 6A). A subgroup analysis was conducted due to high heterogeneity. The results showed that lower frequency VNS (< 25 Hz) (MD = 3.59, 95% CI = 1.97–5.51) was more effective than higher frequency VNS (≥25 Hz) (MD = 0.17, 95% CI = 0.07–0.27) (Figure 6B). Three articles (39, 43, 44) reported the long-term effect as indicated by the WMFT score in the absence of heterogeneity (i.e., *I*^2^ = 0) and found that the VNS group had significantly higher scores than the control group at 90-day posttreatment (MD = 0.30, 95% CI = 0.19–0.47) (Figure 6A).

#### 3.3.3. Prognosis

**Figure 7:**
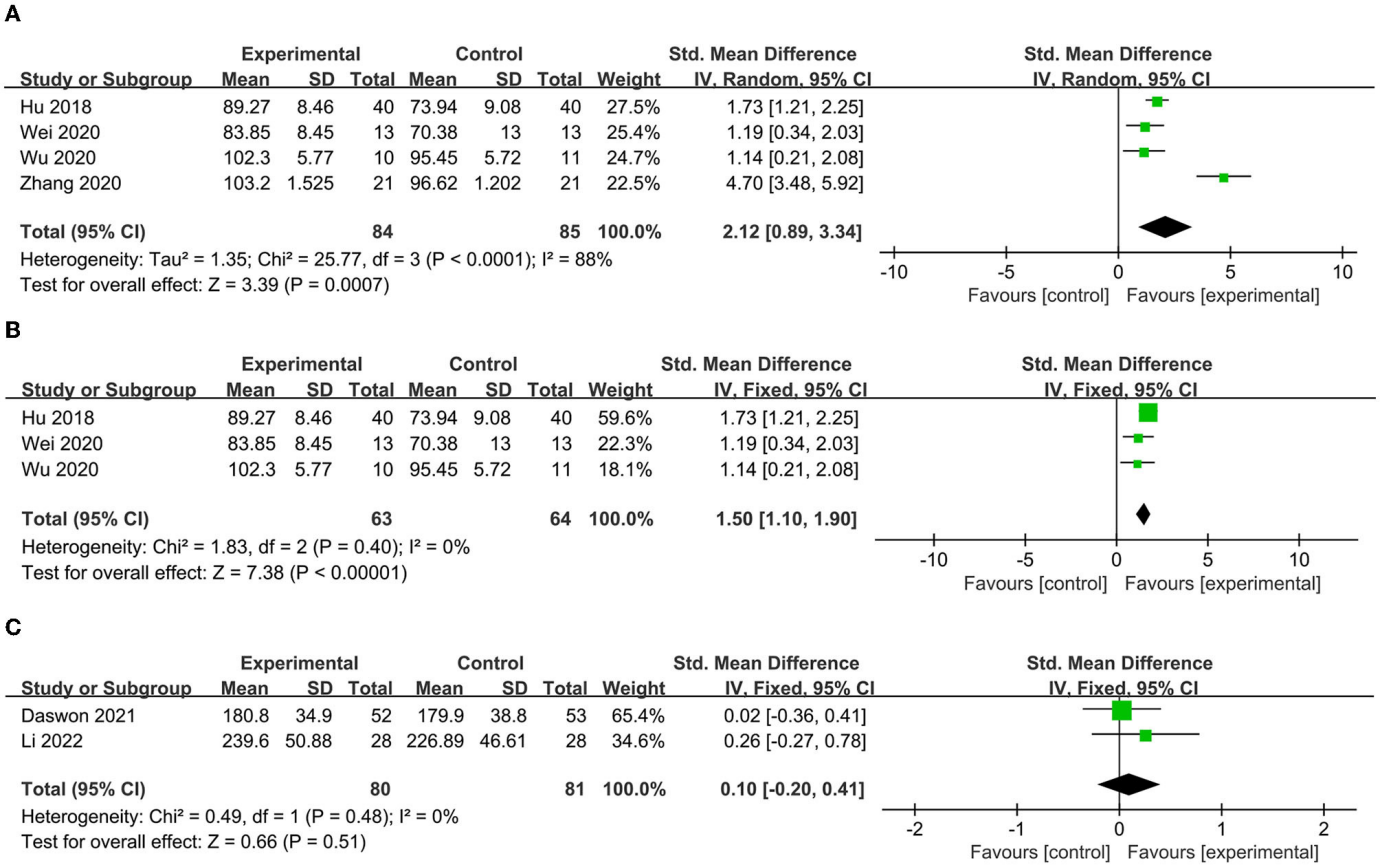
**(A)** Forest map of ADL score before elimination. **(B)** Forest map of ADL score after elimination. **(C)** Forest map of life quality.

To determine the prognosis, we examined the ADL, quality of life, and depression status scores. Four studies (37, 38, 40, 41) included indicators that assessed ADL, including BI, MBI, and FIM. After aggregation, it was found that *I*^2^ was 88% (Figure 7A), indicating excessive heterogeneity. The sensitivity analysis indicated that after excluding the study of Zhang et al. (37), the heterogeneity decreased to 0%. Thus, this indicator was excluded from the analysis. Subsequently, the reanalyzed results indicated that the ADL score was significantly higher after VNS (SMD = 1.50, 95% CI = 1.10–1.90, *I*^2^ = 0%, *p* < 0.00001) (Figure 7B). Two articles (42, 43) included life quality assessment scales, including the Stroke-Specific Quality of Life Scale and EuroQol five-dimensional questionnaire. No significant difference was found between the experimental and control groups after the summary analysis (SMD = 0.10; 95% CI = −0.2 to 0.41, *I*^2^ = 0%; *p* = 0.51) (Figure 7C). Two articles included a scale for assessing depression status, namely, the Beck Depression Inventory (BDI) and the depression domain of the Hospital Anxiety and Depression Scale (HADS). Li et al. (42) found that the HADS score decreased in the experimental group after VNS, while Dawson et al. (43) found a decrease in the BDI score 1 day (−1.6 [SD = 6.2] vs. 0.8 [SD = 5.0]) and 90 days posttreatment (−1.8 [SD = 5.6] vs. 0.2 [SD = 4.1]) compared with that of the control group.

### 3.3. Safety of VNS used in stroke treatment

**Figure 8:**
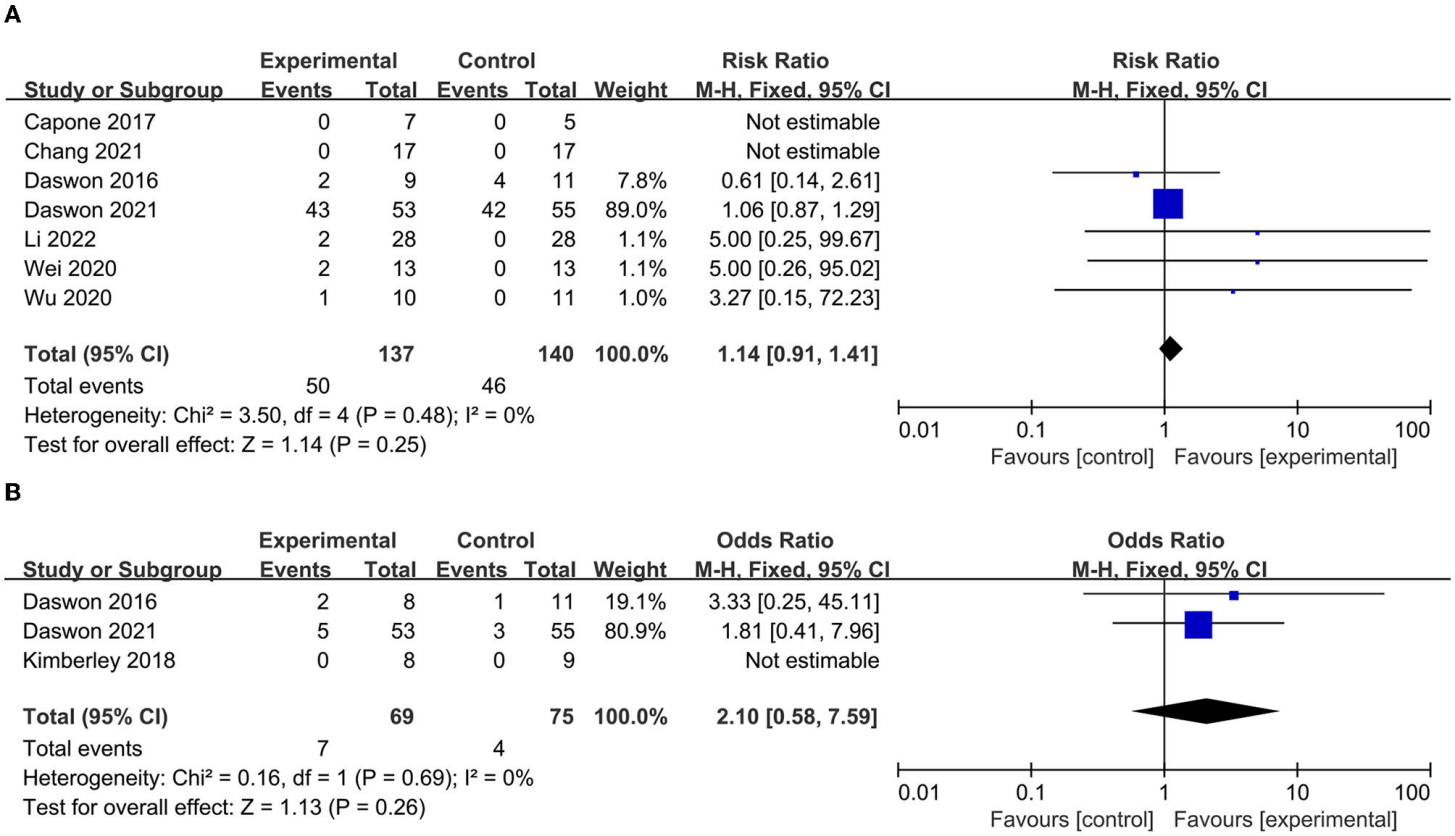
**(A)** Forest plot for the meta-analysis of atrial fibrillations (AEs). **(B)** Forest plot for the meta-analysis of several atrial fibrillations (SAEs).

The AE incidence was reported in seven papers (23, 24, 40–44), and the SAE incidence was reported in three papers (23, 39, 43). As shown in the pooled analysis in Figure 8, there was no significant difference between the experimental and control groups in the incidence of AEs (*p* = 0.25, Figure 8A) or SAEs (*p* = 0.26) (Figure 8B). These results indicate that VNS combined with rehabilitation therapy is safe for the treatment of upper limb dysfunction after stroke.

### 3.4. Publication bias and sensitivity analysis

**Figure 9:**
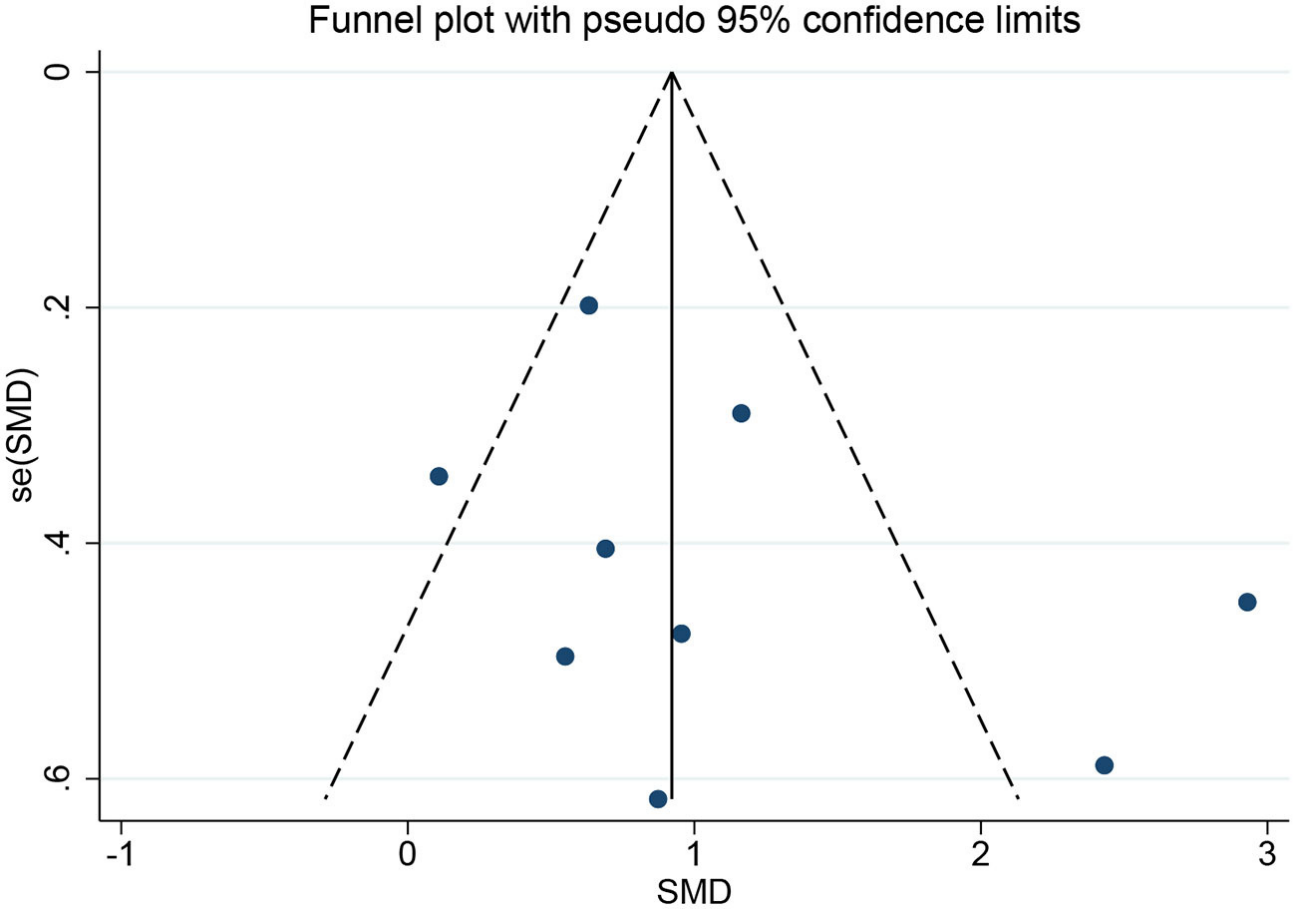
Funnel plots of upper limb motor function.

Quantitative evaluation of the FMA-UE scores using Egger's test indicated no bias (*p* = 0.266). The publication bias chart is shown in Figure 9. Sensitivity analysis was performed by excluding publications one by one. The heterogeneity decreased significantly after removing the study by Chang et al. (44). The bias was presumably related to the intervention duration, which differed from those of other studies. However, Chang et al.'s study did not affect the pooled results or the subgroup analysis results, which were stable.

### 3.5. GRADE quality evaluation

The key outcome indicators (FMA-UE score, ADL score, and the number of AEs and SAEs) of the 10 included studies were evaluated using the GRADE software. The GRADE system evaluates five factors: risk of bias, inconsistency, imprecision, indirectness, and publication bias, and divides the quality of the evidence into four categories: high, medium, low, and very poor. The results according to the GRADE system indicated that the evidence was of high quality for the FMA-UE score, medium quality for the number of AEs and ADL, and low quality for the number of SAEs.

## 4. Discussion

This study included 10 RCTs, which is a larger sample size than the previous meta-analyses that examined the use of VNS in stroke treatment. Publication bias, stimulation parameters, combination regimens, long-term efficacy, and prognosis were integrated and discussed in this study. The results and differences were as follows: (1) Regarding motor function, VNS exerted immediate and long-term effects when combined with comprehensive treatment as indicated by the FMA-UE score, WMFT score, and FMA-UE efficiency, which was consistent with the findings of other meta-analyses. However, the results showed that the FMA-UE pre- and post-treatment difference after 90 days of treatment was lower than that after 1 day of treatment, whereas the WMFT score was the opposite. Therefore, we could only confirm that VNS combined with rehabilitation therapy had a long-term effect and were unable to pinpoint the specific long-term changes. To further investigate this aspect, further clinical studies are warranted. The results of the subgroup analyses suggest that the tVNS mode combined combination therapy and lower frequency of VNS resulted in better outcomes. (2) Prognosis in terms of quality of life was not significant in this study. This result is consistent with the findings of previous studies. In contrast, the increased ability to perform ADL and the remission of depression contradicted the results of Gao et al. (26). For depression, the same two articles were included. The present study used a qualitative analysis considering its excessive heterogeneity. Hence, the effect of VNS on depression needs to be further demonstrated by including future studies. Regarding ADL, Gao et al. (26) included two articles that used the Stroke Impact Scale (hand function) only. In this study, we included more articles and assessed additional indicators, resulting in less heterogeneity. Our results indicated that VNS improved ADL. (3) The present study quantified the occurrence of AEs and SAEs separately to evaluate safety. This has not been considered in previous studies. Based on our results, VNS used in stroke treatment is safe.

Although supported by numerous preclinical and clinical trials, treatment with VNS-targeted plasticity remains challenging due to various factors (6). In the present study, a subgroup analysis of multiple factors was performed. First, we resulted that tVNS was superior to iVNS, which is consistent with the findings of earlier meta-analyses. In this study, all tVNS were taVNS, as tcVNS clinical trials were sparse. Hence, in the future, further investigation of tcVNS is necessary. The results stayed the same after removing the 2016 article by Dawson et al. (23) to eliminate the placebo effect. Second, we conducted a subgroup analysis of the combined VNS protocols, an important factor overlooked in previous meta-analyses, and found that VNS combined with comprehensive training had a better effect on upper limb function than upper limb training alone. We hypothesized that there is a mutually beneficial relationship between different neuroprotective and neuroplastic treatment modalities, suggesting that VNS is a suitable adjunctive therapy for stroke treatment. Furthermore, VNS combined with comprehensive training is recommended in clinical practice. Finally, the ideal parameters for optimizing VNS have long been a highly controversial issue. Optimizing these parameters is crucial for efficacy comparisons (12). One of the major limitations of VNS is its large parameter variations. Variable pulse widths, frequencies, and stimulation currents make it difficult to determine what parameters are more important and which are the best matches or combinations (45). Currently, of the many different parameters, the current intensity is the most studied. Several studies have shown that intensity and plasticity have an inverted U-shape relationship, with medium intensity being superior, and that intensity is inversely proportional to pulse width, with low intensity compensated by wide pulses (46–49). Compared with intensity, frequency is less affected by individualization; therefore, it is easier to optimize, although little research has been conducted to date (10). Buell et al. (50) in their biological experiments verified that frequency and plasticity have an inverted U-shape relationship and that frequencies of ~30 Hz are more effective. Currently, VNS at 20–30 Hz is commonly used in clinical practice. Results from our subgroup analyses demonstrated that VNS at lower frequencies (< 25 Hz) may be more effective than higher frequencies (≥25 Hz), which complements the findings of Buell et al. (50). Additionally, in a study that utilized taVNS for migraines (51), 1 Hz was shown to be more effective than 25 Hz (51). Taken together, these findings imply that lower frequencies may produce superior clinical outcomes. However, owing to diverse clinical applications and the limited number of frequency studies, we cannot exclude the influence of other parameters or factors on frequency. Interestingly, a study (52) has suggested that lower frequencies in tcVNS can be compensated by higher intensities. However, this is inconsistent with our subgroup analysis results on iVNS and taVNS. In the future, more vigilant investigations, including basic experiments and clinical trials, are warranted to verify and validate current findings. In summary, each of these parameters may contribute to the therapeutic effect, and one or more parameters may be altered according to the optimization of the clinical effects in individual patients.

Of note, previous studies on VNS have consistently suggested that the results obtained are influenced by individual differences that inhibit the optimization of stimulation parameters. Theoretically, advanced age is associated with reduced neuroplasticity. Moreover, stroke is dichotomized according to sex and different underlying diseases or drugs may change the effects of VNS through neuroregulatory pathway activation (4). However, preclinical trials have demonstrated that age does not limit the use of VNS in stroke treatment (16); Dawson et al. (53) have conducted further detailed subgroup analyses of their patients after their clinical trials in 2021 and found that differences among different subgroups, including age, sex, residence location, stroke severity, stroke duration, side of the palsy, and cortical involvement, did not affect patient outcomes. An exploratory study by David et al. (54) examined various predictors in combination with two clinical trials and led to the hypothesis that VNS provides additional benefits for patients with more severe upper extremity disability at baseline and unfavorable imaging outcomes (e.g., higher cerebrospinal fluid volume), with no other findings inconsistent with previous speculations. These studies were restricted to specific baseline ranges, and the between-group differences and sample sizes were small. As such, further investigations, especially clinical studies, are needed to justify the above hypothesis and optimize stimulation parameters. Other studies have suggested that relationships exist between stimulation parameters and side effects that may also influence stimulation parameter optimization. These relationships require further clarification. Hence, further studies are needed (55).

This study had some limitations. First, VNS and stroke are both intrinsically heterogeneous situations. Therefore, the variables considered in the subgroup analyses were not entirely homogeneous, which could have interfered with the results. Second, only a few prognostic studies have been published, leading to insufficient evidence for drawing definitive conclusions. Future studies should include a greater number of welldesigned RCTs with high-quality samples. Third, because we did not analyze any objective indicators, future studies should consider the evaluation of neuroimaging and neurophysiological technologies.

## 5. Conclusion

VNS for poststroke upper extremity dysfunction is effective and safe in the long term. It improves upper extremity motor function, increases daily activity capacity, and improves mental state. The results of the subgroup analyses showed that tVNS, combined with integrated rehabilitation and a lower frequency of VNS are superior for the management of poststroke upper extremity function. This study had some limitations that need a comprehensive index and uniform stimulation parameters to further explore the use of VNS in patients with upper limb dysfunction following a stroke.

## Data availability statement

The original contributions presented in the study are included in the article/supplementary material, further inquiries can be directed to the corresponding author.

## Author contributions

QD and YFL accepted accountability for their analysis's accuracy and the data's reliability. The study's inception, design, and manuscript writing were all assisted by XW and WZ. The tables and figures were created by TL and WL. The literature search, data extraction and analysis, data interpretation, and quality assessment were significantly aided by YKL and JY. All authors reviewed and approved the article's submission.
